# Reproductive factors and risk of epithelial ovarian cancer: results from the Asia Cohort Consortium

**DOI:** 10.1038/s41416-024-02924-z

**Published:** 2024-12-20

**Authors:** Melissa A. Merritt, Sarah Krull Abe, Md Rashedul Islam, Md Shafiur Rahman, Eiko Saito, Ryoko Katagiri, Aesun Shin, Ji-Yeob Choi, Loïc Le Marchand, Jeffrey L. Killeen, Yu-Tang Gao, Akiko Tamakoshi, Woon-Puay Koh, Ritsu Sakata, Norie Sawada, Ichiro Tsuji, Yumi Sugawara, Jeongseon Kim, Sue K. Park, Sun-Seog Kweon, Xiao-Ou Shu, Takashi Kimura, Jian-Min Yuan, Shoichiro Tsugane, Seiki Kanemura, Yukai Lu, Min-Ho Shin, Wanqing Wen, Habibul Ahsan, Paolo Boffetta, Kee Seng Chia, Keitaro Matsuo, You-Lin Qiao, Nathaniel Rothman, Wei Zheng, Manami Inoue, Daehee Kang

**Affiliations:** 1 The Daffodil Centre, The University of Sydney, A Joint Venture with Cancer Council New South Wales, Sydney, NSW Australia; 2 Division of Prevention, National Cancer Center Institute for Cancer Control, Tokyo, Japan; 3 Hitotsubashi Institute for Advanced Study, Hitotsubashi University, 2-1 Naka Kunitachi, Tokyo, 186-8601 Japan; 4 Research Center for Child Mental Development, Hamamatsu University School of Medicine, Tokyo, Japan; 5 Sustainable Society Design Center, Graduate School of Frontier Sciences, The University of Tokyo, Tokyo, Japan; 6 Division of Cohort Research, National Cancer Center Institute for Cancer Control, Kashiwa, Japan; 7 National Institute of Health and Nutrition, National Institutes of Biomedical Innovation, Health and Nutrition, Tokyo, Japan; 8 Department of Preventive Medicine, Seoul National University College of Medicine, Seoul, Republic of Korea; 9 Department of Biomedical Sciences, Seoul National University College of Medicine, Seoul, Republic of Korea; 10 Cancer Epidemiology Program, University of Hawaii Cancer Center, Honolulu, HI USA; 11 Department of Pathology, John A. Burns School of Medicine, University of Hawaii, Honolulu, HI USA; 12 Kapiolani Medical Center for Women and Children, Honolulu, HI USA; 13 Department of Epidemiology, Shanghai Cancer Institute, Shanghai, China; 14 Department of Public Health, Hokkaido University Faculty of Medicine, Sapporo, Japan; 15 Healthy Longevity Translational Research Programme, Yong Loo Lin School of Medicine, National University of Singapore, Singapore, 117545 Singapore; 16 Singapore Institute for Clinical Sciences, Agency for Science Technology and Research (A*STAR), Singapore, 117609 Singapore; 17 Radiation Effects Research Foundation, Hiroshima, Japan; 18 Tohoku University Graduate School of Medicine, Miyagi Prefecture, Japan; 19 Graduate School of Cancer Science and Policy, National Cancer Center, Seoul, Republic of Korea; 20 Department of Preventive Medicine, Chonnam National University Medical School, Gwangju, Republic of Korea; 21 Division of Epidemiology, Department of Medicine, Vanderbilt Epidemiology Center, Vanderbilt-Ingram Cancer Center, Vanderbilt University Medical Center, Nashville, TN USA; 22 Division of Cancer Control and Population Sciences, UPMC Hillman Cancer Center, University of Pittsburgh, Pittsburgh, Pennsylvania 15232 USA; 23 Department of Epidemiology, Graduate School of Public Health, University of Pittsburgh, Pittsburgh, PA 15261 USA; 24 Department of Public Health Sciences, University of Chicago, Chicago, IL USA; 25 Stony Brook Cancer Center, Stony Brook University, Stony Brook, NY USA; 26 Department of Medical and Surgical Sciences, University of Bologna, Bologna, Italy; 27 Saw Swee Hock School of Public Health, National University of Singapore, Singapore, Singapore; 28 Division of Cancer Epidemiology and Prevention, Aichi Cancer Center Research Institute, Nagoya, Japan; 29 Department of Cancer Epidemiology, Nagoya University Graduate School of Medicine, Nagoya, Japan; 30 Center for Global Health, School of Population Medicine and Public Health, Chinese Academy of Medical Sciences and Peking Union Medical College, Beijing, China; 31 Division of Cancer Epidemiology and Genetics, Occupational and Environmental Epidemiology Branch, National Cancer Institute, Bethesda, MD USA; 32 Cancer Research Institute, Seoul National University, Seoul, Republic of Korea; 33 Integrated Major in Innovative Medical Science, Seoul National University College of Medicine, Seoul, Republic of Korea

**Keywords:** Risk factors, Ovarian cancer

## Abstract

**Background:**

There are scarce data on risk factors for epithelial ovarian cancer (EOC) in Asian populations. Our goal was to advance knowledge on reproductive -related risk factors for EOC in a large population of Asian women.

**Methods:**

This study used pooled individual data from baseline questionnaires in 11 prospective cohorts (baseline years, 1958–2015) in the Asia Cohort Consortium. A Cox proportional hazards regression model was used to estimate hazard ratios (HRs) and 95% confidence intervals (CIs) adjusting for age, parity and cohort.

**Results:**

After a mean = 17.0 years (SD = 6.3) of follow-up, 674 incident invasive EOC cases were identified among 325,626 women. In multivariable adjusted models we observed an inverse association with parity (5+ children vs. 0, HR = 0.44, 95% CI = 0.28–0.68, Ptrend < 0.001), and a positive association with increasing menopausal age (55+ years vs. <45, HR = 1.77, 95% CI = 1.05–3.01, Ptrend = 0.02) for risk of all EOC.

**Conclusions:**

In this large study of Asian women we identified an inverse association with parity and a positive association with higher menopausal age in relation to EOC risk. Further work is needed to understand EOC risk factors for rare histologic subtypes that occur more frequently in Asian populations.

## Background

There are notable variations in the incidence rates for ovarian cancer by geographic region. Age-standardized incidence rates of ovarian cancer are lower in Asia (5.7–8.5 per 100,000 woman-years in 2003-7) compared with other regions in the world (e.g., 9.6 in US White women and 12.5 in the United Kingdom) [1]. However, a gradual increase in ovarian cancer incidence rates have been observed in Japan (Annual Percent Change = 1.7% between years 1973–77 and 2003–7) as compared with stable rates in Singapore, Thailand and North America over the same time period [1]. A recent study using data from the Korea Central Cancer Registry observed increasing ovarian cancer incidence rates from 1999–2019 [Average Annual Percent Change = 2.3% defining ovarian cancer using the preferred collective definition considering ovarian, fallopian tube and primary peritoneal cancers (*International Classification of Diseases, Tenth Revision*, codes C56, C57 and C48, respectively)] [2]. Similarly, a significant increase in the Average Annual Percent Change for ovarian cancer (C56, 1.5%) has been reported in China from 2000–2018 [3]. Registry based studies lack individual level data on risk factors for ovarian cancer therefore the potential reasons for the observed increasing incidence trends for ovarian cancer in Asia are unknown [4].

The most common type of malignant ovarian cancer, epithelial ovarian cancer (EOC), is a heterogeneous disease that is comprised of at least four major histologic subtypes (serous, endometrioid, clear cell and mucinous). The relative proportions of EOC histologic subtypes differ for several Asian countries; specifically, in Japan, Singapore and Thailand endometrioid, clear cell and mucinous carcinomas comprised a higher proportion of the incidence rate whereas serous carcinomas comprised a lower proportion of the rate as compared with the worldwide distribution (e.g., clear cell carcinoma accounted for 20% of ovarian cancer cases in Japan versus 6% of cases globally) [1]. The observed variation in histologic subtype proportions may be due to differences in exposure to modifiable lifestyle factors and/or genetic contributions to EOC development.

EOC risk factors have been extensively studied but previous study populations mostly represented White women with European ancestry. In contrast, there are few studies on EOC risk factors in Asian women. A previous prospective study in the Singapore Breast Cancer Screening Project evaluated associations between reproductive factors and EOC risk and observed an inverse association with parity and no significant associations with other factors; however, the study was limited by the small number of cases (*n* = 107) [5]. An earlier report in the Shanghai Women’s Health Study (SWHS, representing 174 EOC cases) examined associations for use of different types of contraceptives with risk of ovarian cancer and observed lower EOC risk among long term users (20+ years of use) of IUDs, compared with never use, and no association with oral contraceptive use [6].

In the current study we examined associations between reproductive and hormone-related risk factors with risk of invasive EOC overall and the most common histologic subtype (serous EOC) by analyzing pooled individual-level data from female participants who were enrolled in 11 prospective cohort studies that were conducted in four countries that participate in the Asia Cohort Consortium (ACC). To our knowledge this is the first study to examine the influence of reproductive and hormone-related factors on EOC risk in a large population of Asian women.

## Methods

### Study population

The ACC data collection procedures have been described previously [7]. Briefly, the ACC currently includes 44 participating cohorts from 10 Asian countries and is a collaboration seeking to understand the relationship between genetics, environmental exposures and the etiology of diseases, including cancer, through the establishment of a collection of prospective cohort studies representing at least one million healthy people (https://www.asiacohort.org/index.html).

The current study included 11 ACC cohorts with appropriate information to identify incident EOC cases among subjects who resided in mainland China, Japan, Singapore and South Korea. Most cohorts are population-based studies except for the Korea National Cancer Center (KNCC) cohort, which is a hospital screening center-based study. The source population for the KNCC cohort is participants from the National Cancer Screening Program, provided by the National Health Insurance Services. The National Health Insurance Services is the only health insurance system covering all residents of Korea. Although the recruitment for the KNCC was done in a hospital, the source population fairly represents the general Korean population aged 40 years and older.

From 336,905 female participants, individuals were excluded if they were missing information on age (*n* = 2416); missing extensive data on all of the following reproductive factors (parity status, age at first birth, breastfeeding, oral contraceptive use, age at menarche and menopause, menopausal hormone therapy use, *n* = 6625); reported a prevalent ovarian cancer (*n* = 63); did not have information available on incident ovarian cancer during follow-up (*n* = 1747); subjects who had missing or invalid follow-up data (*n* = 76); participants who were younger than 18 years of age (*n* = 352). After these exclusions, 325,626 women remained in the analysis. Written or oral consent was provided by all subjects who participated in the study. The current study received ethical approval from the executive committee of the ACC and the ethical committee of the National Cancer Center Japan.

### Exposure, covariate and outcome assessment

Reproductive and hormone-related characteristics assessed at the study baseline were harmonized across participating ACC cohorts as detailed previously [8]. Information on reproductive factors, lifestyle characteristics and medical history was collected using a questionnaire at enrollment. Exposure variables included parity (parous women refer to those reporting ≥1 deliveries/children), number of children, age at first delivery, breastfeeding, oral contraceptive (OC) ever use, age at menarche, age at menopause, menopausal hormone therapy (MHT) ever use, Body Mass Index (BMI) and height. Age at menopause and menopausal status were based on self report; information on the cause of menopause was unavailable. When menopausal status was missing, it was assigned using age cutoffs (postmenopausal for ages ≥54 years; premenopausal for ages ≤44 years; ages 45–53 years were left as missing). There were some differences across the cohorts that were noted during harmonization of these reproductive variables. In the LSS cohort, age at first pregnancy was available whereas information on parity status and number of children was not collected; women in LSS were classified as parous if they reported their age at first pregnancy thus the proportion of parous women was likely underestimated. For age at menopause we were unable to distinguish natural from surgical menopause. BMI was analyzed using categories recommended by the World Health Organization for adult Asians [9]. Levels of height were divided into quartiles based on the distribution in the analytic cohort. For the following variables data were only available from selected cohorts as follows: breastfeeding was available in JPHC1, JPHC2, Miyagi, Ohsaki, KMCC, KNCC and Namwon; OC use was available in Miyagi, Ohsaki, KMCC, KNCC, Namwon, SCHS and SWHS; and MHT use was available in Miyagi, Ohsaki, KMCC, KNCC, Namwon, SCHS and SWHS. BMI [7], height and smoking status (ever versus never smoking) at the study baseline were also assessed.

Incident cancer cases were identified by linkage to local cancer registries. Incident invasive EOC cases were defined using the International Classification of Diseases (ICD) 10^th^ revision code C56. International Classification of Diseases for Oncology (ICD-O3) morphology codes were used to censor non-epithelial and non-invasive (borderline) tumors and to define histologic subtypes of EOC (Supplementary Table 1). We evaluated associations for reproductive and hormone-related factors with risk of invasive EOC overall and for the most common histologic subtype (serous/adenocarcinoma not otherwise specified [NOS]).

### Statistical methods

Cox proportional hazards regression using days of follow-up from baseline to the first diagnosis of ovarian cancer or last contact (date of study end, date of death or date of last study contact), whichever occurred first, was used to calculate the hazard ratios (HRs) and 95% confidence intervals (CIs) for the associations between reproductive and hormone-related factors and EOC risk. All models were stratified by the cohort and age at enrollment (5-year age groups: <40, 40–44, to 70–74, 75+ years). Models were adjusted for parity (number of deliveries; 0 [ref], 1-2, 3-4, >4, missing). Analyses of age at first delivery were restricted to parous women with data available on the number of deliveries and these models were adjusted for number of deliveries as follows (1-2 [ref], 3-4, >4). To calculate a *P*-value for the test of linear trend, continuous variables were used when applicable.

Sensitivity analyses were performed to evaluate serous/adenocarcinoma NOS tumors as the outcome. Serous and adenocarcinoma NOS tumors were combined into one category (and are hereafter referred to as serous) because typical serous ovarian adenocarcinoma without other special features (such as mucinous, endometrioid, or clear cell differentiation) may be diagnosed as ‘ovarian adenocarcinoma NOS’ [10]. We examined associations with non-serous histologic subtypes separately but it was necessary to combine these rarer histological subtypes (clear cell, endometrioid, mucinous and other epithelial histologies) because of the small number of cases. To test for heterogeneity in the risk associations between cohorts, data analyses were conducted separately by country and were pooled using meta-analyses random effects models [11]. We did not observe significant heterogeneity in the risk associations between countries therefore all analyses were carried out using pooled data in the entire ACC study population. The proportional hazards assumption was verified using the Grambsch and Therneau method [12]. All statistical tests were two-sided and a *P* < 0.05 was considered statistically significant. Cox proportional hazards analyses and meta-analyses were performed using the ‘survival’ [13] and ‘rmeta’ packages [14], respectively, in R version 4.2.0 [15].

## Results

**Fig. 1:**
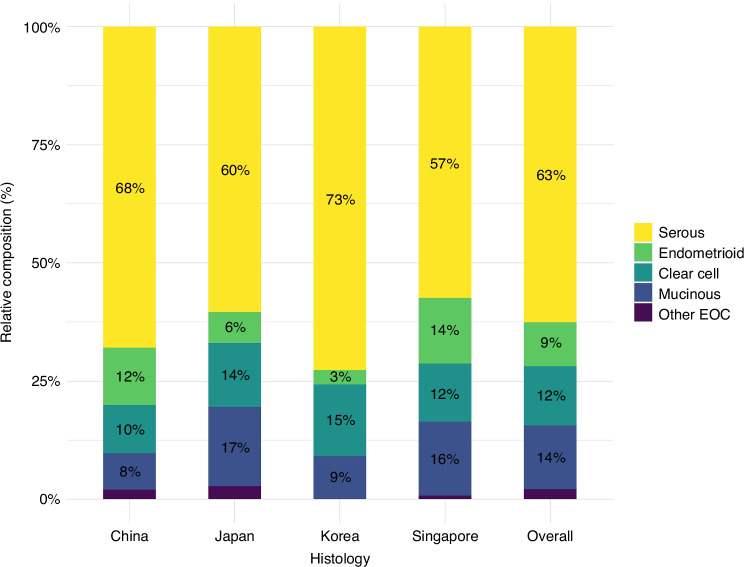
Histogram showing the relative proportion for each histological subtype of epithelial ovarian cancer overall and by country. The proportions for the category “other epithelial ovarian cancer (EOC)” were: 2% (China); 3% (Japan); 0 (Korea); 1% (Singapore); and 2% (overall).

In the ACC study population 674 incident invasive EOC cases (including 422 serous cases) were identified after a mean follow-up of 17.0 years (SD = 6.3). The distribution by histologic subtype for invasive EOC in the ACC overall was 63% serous, 9% (*n* = 63) endometrioid, 14% (*n* = 91) mucinous, 12% (*n* = 84) clear cell and 2% (*n* = 14) other EOC histology (Fig. 1). Most of the ACC cohorts recruited participants in the 1990’s and early 2000’s; exceptions were LSS (the baseline survey was in 1958) and KNCC (the baseline survey was from 2007–15) (Table 1). The mean participant age at the study baseline was 54.2 years (SD = 10.2) and the study included premenopausal and postmenopausal women. The proportion of parous women was high across most studies (93.5% of participants were classified as parous except for LSS which reported only 70.7% parous women). Data on OC use were available in seven cohorts and the proportion of women reporting OC use varied considerably by country; in the two cohorts based in Japan, <5% of women reported OC use whereas the percentage of OC use was higher in cohorts from China (20.4%), Korea (20.7–32.9%) and Singapore (26.4%). Other factors such as the mean age at menarche and menopause, BMI and height were similar across cohorts.

**Table 1:** Characteristics of participants in the Asia Cohort Consortium epithelial ovarian cancer (EOC) study.

	Overall (*n* = 325,626)	SWHS^a^ (*n* = 74,937)	JPHC1^a^ (*n* = 22,011)	JPHC2^a^ (*n* = 29,363)	JACC (*n* = 48,246)	Miyagi (*n* = 24,739)	Ohsaki (*n* = 24,228)	LSS^a^ (*n* = 30,050)	KMCC (*n* = 11,284)	KNCC (*n* = 19,080)	Namwon (*n* = 6385)	SCHS^a^ (*n* = 35,303)
		China	Japan	Japan	Japan	Japan	Japan	Japan	Korea	Korea	Korea	Singapore
Baseline survey, years		1996–2000	1990–92	1992–95	1988–90	1990	1995	1958	1993–2005	2007–15	2004–7	1993–99
EOC cases, *n*	674	196	59	58	22	65	24	95	9	19	5	122
Ovarian case, *n* (missing histology)^b^	133	45	8	11	28	13	15	2	3	0	0	8
**Means (SD)**												
Follow up period, years	17.0 (6.3)	17.4 (3.1)	21.5 (3.7)	18.3 (3.5)	16.3 (5.6)	22.1 (5.5)	10.9 (4.2)	23.3 (10.3)	14.5 (4.3)	9.1 (3.3)	12.7 (2.1)	14.3 (3.5)
Age at enrollment, years	54.2 (10.2)	52.1 (9.1)	49.7 (5.9)	54.5 (8.8)	57.5 (9.9)	52.4 (7.4)	60.7 (10.0)	51.6 (15.1)	55.2 (12.7)	49.8 (9.1)	60.8 (7.9)	56.3 (8.0)
Age at menarche, years^c^	15.0 (1.8)	14.9 (1.7)	14.7 (1.8)	14.9 (2.0)	15.0 (1.8)	14.8 (2.0)	15.3 (1.7)	14.9 (1.7)	16.6 (1.9)	14.8 (1.8)	16.6 (1.9)	14.9 (1.3)
Age at menopause, years^c,d^	48.4 (4.8)	48.0 (4.4)	48.0 (4.7)	48.4 (4.8)	48.6 (4.7)	47.7 (5.6)	48.5 (5.0)	47.8 (4.8)	47.7 (5.5)	48.9 (5.1)	47.5 (5.7)	49.4 (3.9)
Height, cm	153.7 (6.3)	157.5 (5.5)	151.6 (5.3)	151.8 (5.7)	151.0 (5.8)	152.3 (5.5)	151.3 (5.9)	151.5 (6.2)	152.1 (6.0)	157.6 (5.3)	151.7 (5.6)	154.8 (5.8)
Body mass index, kg/m²	23.4 (3.4)	24.0 (3.4)	23.6 (3.1)	23.4 (3.2)	22.9 (3.1)	23.7 (3.1)	23.7 (3.3)	22.0 (3.7)	24.0 (3.4)	23.1 (3.0)	24.6 (3.2)	23.2 (3.3)
**Percentages**												
Premenopausal, %	33.3	49.5	44.2	31.7	13.1	37.7	16.6	42.6	3.2	40.1	11.9	26.6
Postmenopausal, %	66.7	50.5	55.8	68.3	86.9	62.3	83.4	57.4	96.8	59.9	88.1	73.4
Nulliparous^e^, %	6.5	3.3	5.5	5.8	3.9	2.5	3.3	29.3	2.7	3.4	0.6	7.1
Parous, %	93.5	96.7	94.5	94.2	96.1	97.5	96.7	70.7	97.3	96.6	99.4	92.9
1-2 children, %^c^	50.3	75.7	42.1	40.6	44.4	48.4	41.2		22.7	72.3	13.4	28.1
3-4 children, %^c^	34.0	16.7	43.5	39.7	43.5	45.2	45.2		38.0	22.4	42.8	37.0
5+ children, %^c^	11.5	4.3	8.9	13.7	8.2	3.9	10.3		36.6	1.9	43.2	27.8
Never breastfed, %^f^	13.3		14.2	11.4		18.6	14.4		4.3	16.3	2.8	
Ever breastfed, %^f^	86.7		85.8	88.6		81.4	85.6		95.7	83.7	97.2	
Never OC use, %	81.6	79.6				95.6	95.3		67.1	79.0	79.3	73.6
Ever OC use, %	18.4	20.4				4.4	4.7		32.9	21.0	20.7	26.4
Never smoked, %^c^	92.3	97.2	92.5	92.3	93.3	88.7	89.2	84.4	91.1	91.5	94.5	91.2
Ever smoked, %^c^	7.7	2.8	7.5	7.7	6.7	11.3	10.8	15.6	8.9	8.5	5.5	8.8

^a^Data on previous diagnosis of ovarian cancer at baseline were unavailable for indicated cohorts. Missing data on prevalent ovarian cancer were 16% and 8% for JACC and KNCC, respectively, and Miyagi, Ohsaki, KMCC and Namwon had no missing data on prevalent ovarian cancer diagnosis.

^b^These additional first incident ovarian cancer cases were identified; however, information on histology (from International Classification of Diseases for Oncology or ICD-O coding) was unavailable or cases were coded as “Neoplasm, malignant [unknown if epithelial]”; therefore these cases were unable to be classified as EOC and were not considered as events.

^c^The indicated variables had ≥5% missing data in the overall ACC study population as follows: age at menarche, 9.0% missing; age at menopause, 9.2% missing; smoking, 6.3% missing; number of children, 12.9% missing. All other variables in this table had <5% missing data. The percentage of missing data on OC use (2.8%) was calculated among seven studies with data available; missing data on breastfeeding (3.6%) was calculated from seven studies with data.

^d^Restricted to postmenopausal women.

^e^The proportion of nulliparous women was 4.2% when the LSS cohort was excluded.

^f^Restricted to parous women.

*ACC* Asia Cohort Consortium, *EOC* epithelial ovarian cancer, *JACC* [39] Japan Collaborative Cohort Study, *JPHC* [40] Japan Public Health Center–based Prospective Study, *KMCC* [41] Korea Multi-center Cancer Cohort, *KNCC* [42] Korean National Cancer Center Cohort, *Miyagi* [43] Miyagi Cohort Study, *OC* oral contraceptive, *Namwon* [44] The Namwon Study, *Ohsaki* [45] Ohsaki National Health Insurance Cohort Study, *LSS* [46] Life Span Study, *SCHS* [47] Singapore Chinese Health Study, *SD* standard deviation, *SWHS* [48] Shanghai Women’s Health Study.

There were inverse associations between parity with risk of invasive EOC overall (parous yes vs. no, HR = 0.61, 95% CI = 0.47–0.79) and we observed a more pronounced lower EOC risk with a higher number of children (e.g., 5+ children vs. 0, HR = 0.44, 95% CI = 0.28–0.68, Ptrend < 0.001) (Table 2). We observed a positive association between age at menopause and risk of EOC (55+ years vs. <45, HR = 1.77, 95% CI = 1.05–3.01, Ptrend = 0.02). There was also a non-significant positive association with increasing height in relation to EOC risk (per 5 cm increase in height, HR = 1.06, 95% CI = 0.99–1.14, Ptrend = 0.09) and a non-significant inverse associations with age at menarche (17+ years vs. <13, 0.76, 95% CI = 0.54–1.08, Ptrend = 0.06). Other factors (age at first delivery, breastfeeding, OC or MHT use, BMI and smoking) were not associated with EOC risk. There was no significant heterogeneity in these risk associations by country (*P* ≥ 0.07).

**Table 2:** Associations between reproductive factors with risk of epithelial ovarian cancer overall in the Asia Cohort Consortium.

Variable	Value	Total, *N*	Cases, *N*	Model HR^a^
Parous	No	20,592	69	1.00 (Ref)
	Yes	293,859	593	**0.61 (0.47–0.79)**
Number of children	0	11,793	41	1.00 (Ref)
	1-2	142,680	326	**0.67 (0.48–0.94)**
	3-4	96,500	148	**0.51 (0.36–0.72)**
	5+	32,622	50	**0.44 (0.28–0.68)**
	P-trend^b^ (incl 0)			**<0.001**
	P-trend^b^ (parous only)			**0.01**
Age at first delivery^c^	≤20 y	28,819	60	1.00 (Ref)
	21–25 y	130,681	229	0.85 (0.63–1.15)
	26–30 y	87,662	188	0.82 (0.59–1.14)
	31+ y	19,135	41	0.73 (0.47–1.11)
	P-trend^b^			0.16
Breastfeeding (parous women only)^d^	Never	15,829	35	1.00 (Ref)
	Ever	103,219	174	0.95 (0.66–1.38)
Age at menarche	<13 y	22,306	60	1.00 (Ref)
	13-14 y	107,121	242	0.91 (0.69–1.22)
	15-16 y	110,035	243	0.98 (0.74–1.32)
	17+ y	56,863	82	0.76 (0.54–1.08)
	P-trend^b^			0.06
Age at menopause^e^	<45 y	29,757	41	1.00 (Ref)
	45–49 y	66,478	115	1.21 (0.83–1.75)
	50–54 y	82,031	146	1.26 (0.87–1.83)
	55+ y	10,222	24	1.77 (1.05–3.01)
	P-trend^b^			0.02
OC use^f^	No use	155,388	357	1.00 (Ref)
	Ever use	35,110	78	0.97 (0.75–1.25)
MHT use^g^	No use	104,168	213	1.00 (Ref)
	Ever use	9623	11	0.78 (0.42–1.46)
BMI	<18.5 kg/m^2^	16,672	31	0.85 (0.58–1.23)
	18.5–22.9 kg/m^2^	136,021	293	1.00 (Ref)
	23–24.9 kg/m^2^	78,267	155	0.91 (0.75–1.11)
	25–29.9 kg/m^2^	79,182	162	1.02 (0.84–1.24)
	30+ kg/m^2^	11,335	29	1.26 (0.86–1.85)
	Per 5 kg/m^2^ increase			1.05 (0.94–1.18)
	P-trend^b^			0.39
Height	≤149.9 cm	74,398	124	1.00 (Ref)
	>149.9–153.0 cm	84,839	155	0.93 (0.73–1.18)
	>153.0–157.9 cm	78,677	176	1.07 (0.84–1.36)
	>157.9 cm	83,838	215	1.16 (0.91–1.49)
	Per 5 cm increase			1.06 (0.99–1.14)
	P-trend^b^			0.09
Smoking	Never smoked	281,845	601	1.00 (Ref)
	Ever smoked	23,360	52	1.07 (0.80–1.43)

^a^Models were stratified by the cohort and age at enrollment (5-year age groups: <40, 40–44, to 70–74, 75+ years) and were adjusted for parity (number of children; 0 [ref], 1-2, 3-4, >4, missing).

^b^P-trend is the *P*-value for the test of linear trend using continuous variables.

^c^Age at first delivery was restricted to parous women who had data on number of deliveries and these models were adjusted for number of deliveries as follows (1-2 [ref], 3-4, >4). LSS was not included due to missing data on number of deliveries.

^d^Data on breastfeeding were only available in JPHC1, JPHC2, Miyagi, Ohsaki, KMCC, KNCC and Namwon.

^e^Analyses of age at menopause was restricted to postmenopausal women.

^f^Data on OC use were only available in Miyagi, Ohsaki, KMCC, KNCC, Namwon, SCHS and SWHS.

^g^Data on MHT use were only available in Miyagi, Ohsaki, KMCC, KNCC, Namwon, SCHS and SWHS.

*BMI* body mass index, *MHT* menopausal hormone therapy, *OC* oral contraceptive.

Bold values indicate statistically significant results.

We next evaluated the same risk associations for serous (Table 3) and non-serous (all other histological subtypes) EOC (Table 4). In analysis of serous EOC, there was a similar inverse association with parity (parous yes vs. no, HR = 0.67, 95% CI = 0.47–0.95; 5+ children vs. 0, HR = 0.43, 95% CI = 0.25–0.75, Ptrend = 0.001) and a similar non-significant positive association with height (per 5 cm increase in height, HR = 1.07, 95% CI = 0.98–1.17, Ptrend = 0.13). The associations between ages at menopause or menarche with risk of serous EOC were attenuated (e.g., age at menopause, 55+ years vs. <45, HR = 1.15, 95% CI = 0.59–2.23, Ptrend = 0.23). Compared with serous EOC, analyses of non-serous EOC highlighted some differences in risk factor associations. Specifically, there was a lower non-serous EOC risk with a later age at menarche (17+ years vs. <13, HR = 0.54, 95% CI = 0.30–0.97, Ptrend = 0.10) and a higher risk with a later age at menopause (55+ years vs. <45, HR = 4.65, 95% CI = 1.75–12.37, Ptrend = 0.01). A non-significant positive association with higher BMI was also observed (for each 5 kg/m2 increase, HR = 1.20, 95% CI = 1.00–1.44, Ptrend = 0.05) while the association with height was attenuated (per 5 cm increase in height, HR = 1.05, 95% CI = 0.94–1.18, Ptrend = 0.41). Similar to serous EOC, there was an inverse association for parity with risk of non-serous EOC (parous yes vs. no, HR = 0.53, 95% CI = 0.35–0.78).

**Table 3:** Associations between reproductive factors with risk of serous epithelial ovarian cancer in the Asia Cohort Consortium.

Variable	Value	Total	Cases	Model HR^a^
Parous	No	20,592	39	1.00 (Ref)
	Yes	293,859	375	**0.67 (0.47–0.95)**
Number of children	0	11,793	24	1.00 (Ref)
	1-2	142,680	203	0.69 (0.45–1.07)
	3-4	96,500	94	**0.53 (0.33–0.83)**
	5+	32,622	33	**0.43 (0.25–0.75)**
	P-trend^b^ (incl 0)			**0.001**
	P-trend^b^ (parous only)			**0.02**
Age at first delivery^c^	≤20 y	28,819	42	1.00 (Ref)
	21–25 y	130,681	141	0.78 (0.54–1.13)
	26–30 y	87,662	119	0.77 (0.52–1.15)
	31+ y	19,135	23	0.61 (0.35–1.05)
	P-trend^b^			0.07
Breastfeeding (parous women only)^d^	Never	15,829	22	1.00 (Ref)
	Ever	103,219	102	0.81 (0.50–1.30)
Age at menarche	<13 y	22,306	31	1.00 (Ref)
	13-14 y	107,121	149	1.03 (0.70–1.52)
	15-16 y	110,035	151	1.07 (0.72–1.59)
	17+ y	56,863	60	0.93 (0.59–1.47)
	P-trend^b^			0.29
Age at menopause^e^	<45 y	29,757	33	1.00 (Ref)
	45–49 y	66,478	80	1.05 (0.69–1.60)
	50–54 y	82,031	99	1.08 (0.71–1.64)
	55+ y	10,222	13	1.15 (0.59–2.23)
	P-trend^b^			0.23
OC use^f^	No use	155,388	228	1.00 (Ref)
	Ever use	35,110	51	1.02 (0.74–1.41)
MHT use^g^	No use	104,168	150	1.00 (Ref)
	Ever use	9623	7	0.76 (0.35–1.64)
BMI	<18.5 kg/m2	16,672	20	0.85 (0.53–1.36)
	18.5–22.9 kg/m2	136,021	186	1.00 (Ref)
	23–24.9 kg/m2	78,267	99	0.90 (0.70–1.15)
	25–29.9 kg/m2	79,182	99	0.94 (0.73–1.20)
	30+ kg/m2	11,335	16	1.04 (0.62–1.74)
	Per 5 kg/m2 increase			0.97 (0.83–1.12)
	P-trend^b^			0.67
Height	≤149.9 cm	74,398	73	1.00 (Ref)
	>149.9–153.0 cm	84,839	103	1.10 (0.81–1.49)
	>153.0–157.9 cm	78,677	114	1.24 (0.91–1.69)
	>157.9 cm	83,838	130	1.26 (0.91–1.74)
	Per 5 cm increase			1.07 (0.98–1.17)
	P-trend^b^			0.13
Smoking	Never smoked	281,845	373	1.00 (Ref)
	Ever smoked	23,360	34	1.14 (0.79–1.63)

^a^Models were stratified by the cohort and age at enrollment (5-year age groups: <40, 40–44, to 70–74, 75+ years) and were adjusted for parity (number of children; 0 [ref], 1-2, 3-4, >4, missing).

^b^P-trend is the *P*-value for the test of linear trend using continuous variables.

^c^Age at first delivery was restricted to parous women who had data on number of deliveries and these models were adjusted for number of deliveries as follows (1-2 [ref], 3-4, >4). LSS was not included due to missing data on number of deliveries.

^d^Data on breastfeeding were only available in JPHC1, JPHC2, Miyagi, Ohsaki, KMCC, KNCC and Namwon.

^e^Analyses of age at menopause was restricted to postmenopausal women.

^f^Data on OC use were only available in Miyagi, Ohsaki, KMCC, KNCC, Namwon, SCHS and SWHS.

^g^Data on MHT use were only available in Miyagi, Ohsaki, KMCC, KNCC, Namwon, SCHS and SWHS.

*BMI* body mass index, *MHT* menopausal hormone therapy, *OC* oral contraceptive.

Bold values indicate statistically significant results.

**Table 4:** Associations between reproductive factors with risk of non-serous epithelial ovarian cancer (endometrioid, clear cell, mucinous and other epithelial histologies) in the Asia Cohort Consortium.

Variable	Value	Total	Cases	Model HR^a^
Parous	No	20,592	30	1.00 (Ref)
	Yes	293,859	218	**0.53 (0.35–0.78)**
Number of children	0	11,793	17	1.00 (Ref)
	1-2	142,680	123	0.65 (0.39–1.09)
	3-4	96,500	54	**0.48 (0.28–0.83)**
	5+	32,622	17	**0.46 (0.23–0.92)**
	P-trend^b^ (incl 0)			**0.02**
	P-trend^b^ (parous only)			0.22
Age at first delivery^c^	≤20 y	28,819	18	1.00 (Ref)
	21–25 y	130,681	88	1.00 (0.59–1.71)
	26–30 y	87,662	69	0.94 (0.53–1.66)
	31+ y	19,135	18	0.98 (0.48–1.97)
	P–trend^b^			0.98
Breastfeeding (parous women only)^d^	Never	15,829	13	1.00 (Ref)
	Ever	103,219	72	1.19 (0.65–2.18)
Age at menarche	<13 y	22,306	29	1.00 (Ref)
	13-14 y	107,121	93	0.79 (0.52–1.20)
	15-16 y	110,035	92	0.90 (0.59–1.39)
	17+ y	56,863	22	**0.54 (0.30–0.97)**
	P–trend^b^			0.10
Age at menopause^e^	<45 y	29,757	8	1.00 (Ref)
	45–49 y	66,478	35	1.87 (0.83–4.24)
	50–54 y	82,031	47	2.03 (0.90–4.58)
	55+ yrs	10,222	11	**4.65 (1.75–12.37)**
	P-trend^b^			**0.01**
OC use^f^	No use	155,388	129	1.00 (Ref)
	Ever use	35,110	27	0.88 (0.57–1.36)
MHT use^g^	No use	104,168	63	1.00 (Ref)
	Ever use	9623	4	0.84 (0.30–2.39)
BMI	<18.5 kg/m2	16,672	11	0.83 (0.45–1.56)
	18.5–22.9 kg/m2	136,021	107	1.00 (Ref)
	23–24.9 kg/m2	78,267	56	0.92 (0.67–1.28)
	25–29.9 kg/m2	79,182	63	1.17 (0.85–1.61)
	30+ kg/m2	11,335	13	1.69 (0.94–3.02)
	Per 5 kg/m2 increase			1.20 (1.00–1.44)
	P-trend^b^			**0.05**
Height	≤149.9 cm	74,398	51	1.00 (Ref)
	>149.9–153.0 cm	86,511	52	0.69 (0.46–1.02)
	>153.0–157.9 cm	77,005	62	0.84 (0.57–1.24)
	>157.9 cm	83,838	85	1.02 (0.69–1.51)
	Per 5 cm increase			1.05 (0.94–1.18)
	P-trend^b^			0.41
Smoking	Never smoked	281,845	228	1.00 (Ref)
	Ever smoked	23,360	18	0.96 (0.59–1.56)

^a^Models were stratified by the cohort and age at enrollment (5-year age groups: <40, 40–44, to 70–74, 75+ years) and were adjusted for parity (number of children; 0 [ref], 1-2, 3-4, >4, missing).

^b^P-trend is the *P*-value for the test of linear trend using continuous variables.

^c^Age at first delivery was restricted to parous women who had data on number of deliveries and these models were adjusted for number of deliveries as follows (1-2 [ref], 3-4, >4). LSS was not included due to missing data on number of deliveries.

^d^Data on breastfeeding were only available in JPHC1, JPHC2, Miyagi, Ohsaki, KMCC, KNCC and Namwon.

^e^Analyses of age at menopause was restricted to postmenopausal women.

^f^Data on OC use were only available in Miyagi, Ohsaki, KMCC, KNCC, Namwon, SCHS and SWHS.

^g^Data on MHT use were only available in Miyagi, Ohsaki, KMCC, KNCC, Namwon, SCHS and SWHS.

*BMI* body mass index, *MHT* menopausal hormone therapy, *OC* oral contraceptive.

Bold values indicate statistically significant results.

## Discussion

We evaluated a range of reproductive and hormone-related factors in relation to EOC risk in the ACC study population which included data from 11 prospective cohorts from four Asian countries. To our knowledge this was the first large scale study of EOC risk factors in Asian women. In analysis of EOC risk overall, we observed a significant inverse association with parity and a positive association with older age at menopause. There was also a non-significant positive association with height, and a non-significant inverse association with older age at menarche in relation to EOC risk. In analyses by histological subtype, the associations with parity and height were still apparent for serous EOC. Associations with parity, ages at menarche and menopause and a non-significant association with BMI were observed for non-serous EOC.

The inverse association with parity in relation to risk of EOC in the current study is consistent with a previous Ovarian Cancer Cohort Consortium (OC3) analysis which observed that women who had ever versus never had children had a 31% lower risk and a 10% reduction in risk was calculated for each full-term pregnancy (including nulliparous women as the referent group) [16]. The OC3 study analyzed data from 21 prospective cohort studies representing mostly White women in North America, Europe and one study from Asia (Singapore Chinese Health Study, which is included in the current ACC analysis). Several hypotheses have been suggested to explain the inverse association between parity and EOC risk including: anovulation [17]; lower pituitary gonadotrophin secretion [18]; higher levels of progesterone that may promote apoptosis [19]; and the hypothesis that pregnancy may clear away cells that have accumulated somatic mutations that could lead to malignant transformation [20]. Recent investigations using data from a Danish nationwide cohort study [21] and a pooled analysis of 15 case-control studies [22] have provided new insights regarding how pregnancies of shorter duration influence ovarian cancer risk; these studies observed a similar lower EOC risk irrespective of the type of pregnancy (induced abortion, spontaneous abortion and full term pregnancy). To explain this observation Husby et al. [21] proposed that pregnancy may alter the fallopian tube fimbria epithelium, which is thought to be the precursor cell for serous EOC [23, 24], by contributing to a “dormant state” (low proliferative activity) in the fallopian tube epithelium. In the current study we were unable to evaluate associations with incomplete pregnancies because only data on number of deliveries was available.

Our observation that an older age at menopause led to a higher risk of EOC is consistent with the observation of a 6% increase in risk of invasive EOC (95% CI = 1.02–1.10) per 5 years of delayed age at menopause in the OC3 study population [16]. The OC3 analysis reported that a later age at menopause was specifically associated with a higher risk of endometrioid and clear cell carcinomas but not serous carcinoma. We similarly observed that the association with menopausal age was no longer significant for serous EOC and a more pronounced association was observed for non-serous EOC. It was not possible to evaluate specific non-serous histologic subtypes in the current study due to limited case numbers. It has been hypothesized that an older age at menopause may be associated with higher EOC risk because it lengthens the menstrual lifespan and may prolong exposure to endogenous steroid hormones, such as estrogen [25], that could promote EOC development [26, 27]. Having an older age at menarche would also shorten the menstrual lifespan and the current study identified a non-significant 24% lower risk of invasive EOC and a 46% lower risk of non-serous EOC for women who reported an older age at menarche (comparing 17+ years vs. <13). The association with age at menarche was not observed in analysis of serous EOC.

We observed a non-significant positive association between height and EOC risk in the current study. Several previous meta-analyses [28, 29] and two cohort studies in Korea [30, 31] similarly reported a higher risk of ovarian cancer with increasing height. The positive association with height was consistent across different histologic subtypes of EOC [28]. In the current study, non-significant associations with height were only observed in analyses of EOC overall and serous histology. Previous studies using a Mendelian randomization approach observed that individuals of European ancestry with greater predicted height had a higher risk of invasive EOC which supports the potential causal association between increasing height and EOC risk [32]. It appears that links between increasing height with a higher risk of developing cancer is not specific for EOC. A systematic review reported that increasing height was associated with a higher risk of several types of cancer with risk estimates for each 5 cm increment in height ranging from 4% in prostate cancer to 12% for malignant melamona [33]. The biological pathway that underlies the height-cancer risk association is complex because adult height is determined by genetic predisposition as well as a variety of other extrinsic factors including environmental determinants, hormone levels and nutrition [34, 35]. Insulin-like growth factor 1 is of interest in relation to its effects on both height and cancer risk; however, a recent Mendelian randomization study that examined genetically predicted serum IGF-1 levels in participants with European ancestry observed no association with ovarian cancer risk [36].

In the current study there was no association with OC ever versus never use for risk of EOC. This finding concurs with a previous report from the Shanghai Women’s Health Study [6] (included in the current ACC study). Previous reports (mostly representing White women) have consistently reported inverse associations between OC ever versus never use with EOC risk and more pronounced reductions in EOC risk have been observed with a longer duration of use and with more recent OC use [37]. It has been hypothesized that the protective association between OC use on risk of developing EOC may be due to OCs reducing the number of lifetime ovulations and by lowering intra-ovarian estrogen levels [38]. The lack of an association in the current ACC study may be due to the limited data available on OC ever versus never use (these data were available in only seven of the ACC cohorts) and the small number of exposed cases (in the two Japan cohorts the proportion of participants who reported ever use of OCs was <5%). It is also possible that some of the users may have used OCs for a very short duration and this may have attenuated the risk association towards the null value.

This study has several strengths; since data were collected prospectively it was unlikely to be biased by disease status and therefore any misclassification was likely nondifferential and would be expected to attenuate the risk estimates towards the null. There are currently few data on ovarian cancer risk factors in Asian women. As far as we are aware the current study appears to be the largest to date to analyze a range of reproductive and hormone-related factors in relation to EOC risk in an Asian population. Potential limitations included the lack of information on history of oophorectomy at baseline therefore the number of incident EOC cancer cases identified in this study may be lower than expected. We also did not have information on other ovarian cancer risk factors including tubal ligation, endometriosis and family history of ovarian cancer. The current ACC analysis did not evaluate hysterectomy because only two out of eleven cohorts had these data available. Only a single assessment of exposure at the study baseline was available for analysis; however, it is unlikely that many of the reproductive characteristics would change over time particularly for postmenopausal women who represented 67% of the overall study population. For selected factors such as MHT use there may be greater potential for exposure misclassification using only the baseline exposure assessment. Although data were pooled across 11 cohorts to increase the sample size for the current analysis, there were still limited numbers of non-serous cases and we were unable to explore risk factors for specific rare EOC histological subtypes. Since clear cell, endometrioid and mucinous histologic subtypes comprise a larger proportion of EOCs in Asia [1] it will be important to evaluate risk factor associations for these histologic subtypes of EOC among Asian women in future studies.

To our knowledge this is the first large-scale study of a range of risk factors for EOC in an Asian population which has historically reported low incidence rates of EOC as compared to other parts of the world. This study identified an inverse association with parity and a positive association with age at menopause in relation to risk of EOC. There were also non-significant inverse associations for an older vs. younger age at menarche for risk of EOC. Associations with ages at menarche and menopause were more pronounced for non-serous EOC. Since there are differences in the distribution of EOC risk factors in Asian populations, it will be of interest to compare the population attributable fractions for established EOC risk factors in Asia compared with populations outside of Asia. Further work is also needed to study EOC risk factors in Asian populations with a focus on rare EOC histological subtypes when possible and to evaluate risk factors (such as endometriosis) that were not available in the current study. The ultimate goal of this work is to understand EOC risk factors in order to contribute to the development of improved measures for EOC prevention.

## Supplementary information

Supplementary material

STROBE checklist

## Data Availability

Investigators are granted access to the Asia Cohort Consortium data upon reasonable request and with the approval of ACC members and the Institutional Review Board. We cannot publicly provide individual data due to participant privacy based on the ethical guidelines in Japan. Additionally, the informed consent that the participating studies obtained did not include a provision for publicly sharing data. Data described in the manuscript, code book, and analytic code are available from the corresponding author upon request.
