# Supplementary material for: Reproductive factors and risk of epithelial ovarian cancer: results from the Asia Cohort Consortium
Source: Br J Cancer. 2024 Dec 20;132(4):361–70. doi: 10.1038/s41416-024-02924-z (PMC11833059; doi:10.1038/s41416-024-02924-z)
Supplement: Supplementary file 1 — Supplementary material [file 41416_2024_2924_MOESM1_ESM.docx]

Merritt *et al*. ‘Reproductive factors and risk of epithelial ovarian cancer: results from the Asia Cohort Consortium’

**Supplementary tables**

**Supplementary Table 1**. International Classification of Diseases for Oncology 2nd or 3rd revision (ICD-O2 and -3, respectively) topography and morphology codes used to define invasive epithelial ovarian cancer and histological subtypes in participating cohorts in the Asia Cohort Consortium.

| **ICD-O2/-O3 topography code**  Include*:* C56 | **ICD-O2/-O3 morphology codes**  Censor*:* 8000, 8001, 8060, 8070, 8032, 8041, 8070, 8320, 8442, 8490, 8500, 8620, 8800, 9060, 9080, 9081, 9084, 9580, 9581 |
| --- | --- |

| **Tumor histology classification** | **ICD-O2/-O3 morphology codes** |
| --- | --- |
| Serous | 8020 |
|  | 8120 |
|  | 8260 |
|  | 8441 |
|  | 8450 |
|  | 8460 |
|  | 8461 |
|  | 8462 |
|  | 8504 |
| Endometrioid | 8380 |
|  | 8381 |
|  | 8560 |
| Clear cell | 8310 |
|  | 8313 |
| Mucinous | 8470 |
|  | 8471 |
|  | 8472 |
|  | 8473 |
|  | 8480 |
|  | 8481 |
|  | 8482 |
| Adenocarcinoma not otherwise specified (NOS) | 8010 |
|  | 8140 |
|  | 8440 |
| Other specified epithelial ovarian cancer (including carcinosarcoma) | 8240 |
|  | 8323 |
|  | 8950 |
|  | 8980 |
|  | 8951 |
|  | 9000 |
